# Medical students’ attitudes toward providing patients with audio recordings of their medical encounters: a cross-sectional online survey

**DOI:** 10.1186/s12909-025-07460-9

**Published:** 2025-06-19

**Authors:** Cheyenne Topf, Marina Buzo, Pola Hahlweg, Isabelle Scholl

**Affiliations:** Department of Medical Psychology, University Medical Center Hamburg-Eppendorf, Hamburg, Germany

**Keywords:** Consultation recordings, Cross-sectional online study, Medical students, Patient-centered care, Patient information, Patient communication, Patient-physician-relationship

## Abstract

**Background:**

Medical encounters often involve complex information that can be challenging to process, especially in emotionally charged situations. Providing patients with audio recordings of their medical encounters, termed consultation recordings, has shown promising benefits such as improved information recall and understanding. In prior research, patients largely reported favorable attitudes toward consultation recordings; physicians were more skeptical, particularly regarding legal risks. To date, consultation recordings are rare in Germany. This study aimed at understanding medical students’ attitudes toward consultation recordings, as they are the healthcare professionals of the future and could therewith play a crucial role in further implementation efforts.

**Methods:**

We conducted a nationwide cross-sectional quantitative online survey with medical students in Germany, assessing attitudes toward and desire for future consultation recordings. Data was analyzed using descriptive statistics.

**Results:**

Two hundred twenty-two participants were included. 56% of participants expressed positive attitudes toward consultation recordings, acknowledging benefits such as information recall and preparation for follow-up consultations. However, they also expressed significant concerns about potential misuse, legal implications, and physicians feeling pressured. About 16% of participants expressed a clear willingness to offer consultation recordings in the future, while 44% were hesitant.

**Conclusions:**

Our findings that medical students recognize potential benefits of consultation recordings, while simultaneously expressing concerns about this intervention, are comparable to results from studies conducted in physician samples. Medical students’ limited clinical experience and the low prevalence of consultation recordings in Germany likely contribute to these apprehensions. These mixed attitudes could be addressed by providing opportunities for positive experiences with consultation recordings during medical and postgraduate education as well as in clinical practice. Providing role models who support patient-centered care could also foster greater acceptance. Future research should focus on the integration of patient-centered interventions such as consultation recordings in the medical curricula. Overall, consultation recordings have the potential to become a valuable tool in routine healthcare if current barriers are effectively addressed.

**Supplementary Information:**

The online version contains supplementary material available at 10.1186/s12909-025-07460-9.

## Background

Medical encounters often involve the communication of complex and extensive information, which can be challenging for patients to process and retain [1–3]. This challenge becomes particularly pronounced when patients experience strong emotional reactions such as shock, numbness, anxiety or anger following the diagnosis of a severe disease [1, 2, 4]. Such states interfere with their cognitive processing of medical information [1, 2]. Research further shows that patients forget up to 80% of the information provided during medical encounters [1, 5, 6]. Ensuring that patients receive comprehensible information is not only a clinical priority but also emphasized in German and international health policy [7, 8]. Beyond that, research has demonstrated that patients with fulfilled information needs experience better health-related quality of life and less anxiety and depression [9]. An internationally well-researched approach to addressing those issues is providing patients with audio recordings of their medical encounters, often referred to as consultation recordings [2, 5, 10, 11]. Consultation recordings have shown to offer a great number of benefits, including aiding of information recall [2, 5, 10–15], enhancing of understanding [10, 12, 14, 16], and increasing patients’ sense of being informed [11, 13, 17]. Beyond those informational aspects, they can also empower patients [14, 15, 18–20], reduce anxiety and depression [2, 11, 21], facilitate the sharing and discussion of medical information with family members [13, 14, 19, 21], improve the treatment decision-making process [11, 13, 14, 21], and contribute to patients’ overall satisfaction with care [13]. While several studies found that most patients expressed positive attitudes toward consultation recordings [10, 13, 15, 17, 21–23], research also revealed that physicians often remain apprehensive [10, 11, 16, 22, 24–27]. They do acknowledge the benefits of consultation recordings [10, 16, 24–27], but simultaneously worry about the legal implications of consultation recordings [10, 16, 22, 24–26], including confidentiality and data protection [22, 24], as well as about the risk of misuse of those recordings [16, 22, 24, 26]. There is also concern regarding potential negative impacts on the clinician-patient relationship [10, 16, 22, 25, 26], that recordings could escalate patient anxiety [25], or that recordings might prolong consultations [2, 22, 25]. Consultation recordings can be implemented in various ways, such as patient-led recordings using own devices, covert recordings, or provider-led recordings offered by healthcare services or through smartphone apps [10, 18, 19, 28, 29]. Internationally, consultation recordings remain underutilized. In two previous studies in Germany, only 5% of patients with cancer, and around 12% of oncologists reported experiences with consultation recordings [23, 30]. In contrast, studies from various countries, including the United Kingdom and the United States, indicated that 15 [18] to 18 [10] percent of patients and up to 93% of physicians reported having recorded medical encounters in the past [10, 16, 26, 31]. The limited prevalence of consultation recordings might be linked to the described provider concerns. International research suggests that past positive encounters with such recordings can shape physicians’ view on consultation recordings [11, 16, 26]. It is essential to address physicians’ concerns to facilitate wider acceptance and implementation of consultation recordings in clinical practice, as their support is critical for integrating this intervention into routine care [16, 32]. Understanding the perspective of future healthcare professionals, such as medical students, is particularly important, as they will soon become practicing physicians who may encounter and implement consultation recordings in their own clinical practice. Up to this date, the investigation of medical students’ views on consultation recordings remains underexplored. In this study we aimed to assess medical students’ attitudes toward consultation recordings.

## Methods

We report on a cross-sectional nationwide quantitative online survey exploring attitudes toward consultation recordings of medical students in Germany. Reporting follows the Checklist for Reporting Results of Internet E-Surveys (CHERRIES) [33] (cp. Supplementary File 1). The methodology of this study was based on that of a previous study we conducted with physicians in oncology [30].

### Participants

Medical students enrolled at a German university were eligible to participate. No further inclusion or exclusion criteria applied. With a population size of 105,275 medical students enrolled at a German university in the winter semester of 2021 [34], a confidence level of 0.85, and a margin of error of 0.05, the estimated minimal sample size was calculated as 207 (calculated with www.surveymonkey.com/mp/sample-size-calculator/). However, as we used convenience sampling instead of a random sample, representativeness and generalizability have to be considered with caution (see limitation section).

### Materials and questionnaires

The development of the quantitative questionnaire was based on the quantitative questionnaire of a previous study we conducted with physicians in oncology [30]. The questionnaire was adapted to the population of medical students (including questions regarding the progress in the medical education program and previous clinical experience). The survey assessed participants’ attitudes toward and desire for consultation recordings. The adaptation of the questionnaire was pretested with medical students working at our department (*n* = 2). The final survey can be found in Supplementary File 2.

The first pages of the survey included an informed consent form and a screening question. In the first section of the survey we assessed the participants’ general attitude toward consultation recordings using three items (see Supplementary File 2 for items). Each item was scored on a 6-point Likert scale, ranging from *does not apply at all (= 1)* to *fully applies (= 6)* or from *very negative (= 1)* to *very positive (= 6).* Additionally, participants evaluated 50 statements regarding potential benefits and concerns related to consultation recordings on a 6-point Likert scale ranging from *completely disagree (= 1)* to *completely agree (= 6)*. We provided a “no response” option for the items on attitudes, potential benefits, and concerns, in case participants would be unable to assess certain statements, as they were evaluating an intervention with which they had no prior experience. The second section focused on participants’ desire for future consultation recordings. Those who indicated openness to record medical encounters in the future were asked follow-up questions (e.g., “Would you be open to patients recording on their cell phone?”). For one item, participants were able to further specify their answer in an open format (to specify the willingness to listen to consultation recordings).

Participants’ preferred role in treatment decisions was assessed using an adapted version of the Control Preferences Scale (CPS) [35]. This one-item instrument assesses whether participants prefer decision-making to be primarily led by the physician, the patient, or shared between both. Participants’ inclination toward actively engaging with technical systems was measured using the German version of the Affinity for Technology Interaction Short Scale (ATI-S) [36]. ATI-S comprises four items rated on a 6-point Likert scale, ranging from *completely disagree (= 1) to completely agree (= 6)*. The ATI-S demonstrated strong psychometric properties, including high McDonald’s omega, factor loadings, item difficulty and discrimination, and construct validity [36]. Additionally, demographic data (e.g. age, gender), medical education progress (e.g. completed exams and internships), clinical experience (e.g. previous training in another medical profession, patient contact experience), and knowledge of laws regulating audio recordings in Germany were collected. To ensure data quality, the survey included an attention check item to identify careless responses.

### Data collection

We employed a convenience sampling approach and utilized multiple recruitment strategies. We contacted all medical faculties of public and private universities in Germany, asking them to forward the study invitations. Furthermore, we shared invitations via social media and distributed leaflets and posters at various locations across the University Medical Center Hamburg-Eppendorf and the Medical School Hamburg. Participants were required to provide informed consent electronically, before participating in the online survey. They confirmed through self-report that they are currently enrolled at a German university to study human medicine. The survey was conducted between June and July 2023. We utilized the LimeSurvey platform [37]. As an incentive, participants could enter a raffle to win one of 25 vouchers, each valued at 10 euros.

### Data analysis

We included all participants in the analysis who met the inclusion criteria, completed the survey, and answered the attention check item correctly. Missing data was not imputed. The ATI-S was analyzed according to its manual. Open-format specifications to one item were categorized and subsequently included in the analyses. For all items on attitudes, we calculated frequency distributions, means, standard deviations, medians, and interquartile ranges. For items on desire for consultation recordings, we calculated frequency distributions. For sociodemographic data, we primarily calculated frequency distributions. We formulated two hypotheses for subgroup testing, based on previous results that linked female gender with higher patient-centered attitudes [38, 39], and more clinical experience with more positive attitudes toward consultation recordings [26]: (1) The attitude toward consultation recordings is more positive in female medical students than in male students, (2) The attitude toward consultation recordings is more positive in medical students with regular patient contact compared to those without regular patient contact. Regarding hypothesis 1, we compared the groups “Female” and “Male”. We excluded the third group “Non-binary/diverse” from analysis due to small subsample size. Regarding hypothesis 2, we compared the groups “Medical students with regular patient contact” and “Medical students with no regular patient contact”. The Kolmogorov-Smirnov test revealed that the distribution of the attitudes toward consultation recordings differed between the groups “Female” and “Male” (*p* <.05), which is why we report the mean rank in the following analysis [40, 41]. The distribution did not differ between the groups “Medical students with regular patient contact” and “Medical students with no regular patient contact” (*p* >.05), therefor we report the median in the subsequent analysis [40, 41]. The Mann-Whitney-U-Test was used to determine whether there is a statistically significant difference between the subgroups in the ordinal dependent variable (general attitude) [42]. Given the two co-primary hypotheses, the significance level was adjusted to *p* <.025 using the Bonferroni correction [43]. Data were analyzed using SPSS 27.

## Results

### Participant characteristics

Two hundred twenty-six participants met the inclusion criteria and completed the survey. Four participants were excluded for incorrectly responding to the attention check item, leaving 222 participants for analysis. Participants were female in most cases (*n* = 156, 70.3% female; *n* = 63, 28.4% male; *n* = 3, 1.4% non-binary/diverse) and between 18 and 34 years old (X̅=23.22, SD = 3.31). Most of the participants were in their first or second year (*n* = 104, 46.8%) or in their third to fifth year (*n* = 101, 45.5%). A small proportion were in their clinical internship year (sixth year; *n* = 11, 5%) or beyond (*n* = 6, 2.7%). About a quarter had previous training in another medical profession (e.g., nursing or medical assistant; *n* = 57, 25.7%). Of those, around a third had less than one year (*n* = 17, 29.8%) or one to three years (*n* = 17, 29.8%) and around a fifth had four to six years (*n* = 10, 17.5%) or more than seven years (*n* = 13, 22.8%) of clinical experience in this profession. About half of the participants had regular patient contact at the time of the survey (*n* = 110, 49.5%). The sample showed a moderate level of technological affinity (X̅=3.45; SD = 1.10) and most participants did not know the laws regulating audio recordings in Germany (*n* = 200, 90.1%). The vast majority of participants (*n* = 217, 97.8%) preferred either a patient-led (*n* = 123, 55.5%) or a shared (*n* = 94, 42.3%) decision-making style. Additional participant characteristics can be found in Table 1.

**Table 1:** Participant characteristics (*N* = 222)

Participant characteristics (*N* = 222)	
Sex, *n (%)*	
Female	156 (70.3)
Male	63 (28.4)
Non-binary/diverse	3 (1.4)
Age in years, *mean (SD) [min; max]*	23.22 (3.31) [18;34]
Current year in medical education, *n (%)*	
First or second year	104 (46.8)
Third to fifth year	101 (45.5)
Sixth year (Clinical internship year)	11 (5.0)
Seventh year or beyond	6 (2.7)
Completed state medical exams^1^, *n (%)*^2^	
None yet	101 (45.5)
First state medical exam only	106 (47.7)
First and second state medical exam	16 (7.2)
Completed internships^3^, *n (%)*^*4*^	
None yet	4 (1.8)
Nursing internship only^5^	120 (54.1)
Clinical elective^6^	96 (43.2)
Less than 1 month	1 (1.0)
1-2 months	48 (50.0)
3-4 months	39 (40.6)
More than 4 months	8 (8.3)
Clinical internship year^7^	13 (5.9)
Previous training in another medical profession (e.g. nursing, medical assistant), *n (%)*	
No	165 (74.3)
Yes	57 (25.7)
Work experience in this profession	
Less than 1 year	17 (29.8)
1-3 years	17 (29.8)
4-6 years	10 (17.5)
More than 7 years	13 (22.8)
Regular patient contact beyond medical school education, *n (%)*	
No	112 (50.5)
Yes	110 (49.5)
Affinity for Technology Interaction Scale, *mean (SD) [min; max]*	3.45 (1.10)
1 to 6, higher more	[1;6]
Perceived knowledge of laws regulating audio recordings in Germany, *n (%)*^8^	
No	200 (90.1)
Yes	17 (7.7)
Preferred level of involvement in treatment decisions, *n (%)*	
Patients should make the decision what medical treatment they receive.	9 (4.1)
Patients should ultimately make the decision about their medical treatment, after having seriously considered my medical opinion.	114 (51.4)
I prefer my patient and I to share the responsibility for making the decision which medical treatment is best for them.	94 (42.3)
I prefer to make the final decision about the patient’s medical treatment, considering their opinion.	5 (2.3)
As a doctor, I prefer to make all decisions concerning the patient’s medical treatment.	0 (0.0)
Location of medical school (German states)^9,10^, *n (%)*	
North Rhine-Westphalia	93 (41.9)
Hamburg	72 (32.4)
Saarland	13 (5.9)
Baden-Württemberg	11 (5.0)
Saxony-Anhalt	8 (3.6)
Berlin	7 (3.2)
Bavaria	5 (2.3)
Mecklenburg-Western Pomerania	4 (1.8)
Lower Saxony	2 (0.9)
Rhineland-Palatinate	2 (0.9)
Brandenburg	1 (0.5)
Hesse	1 (0.5)
Saxony	1 (0.5)
Schleswig-Holstein	1 (0.5)
Thuringia	1 (0.5)

Notes. ^1^Students are required to pass 3 medical state exams (German: *Staatsexamen*) in total; ^2^A participant has incorrectly selected both “none yet” and “first state medical exam,” which is why the frequency and percentage are higher here; ^3^Students who have completed the clinical internship year have also completed the nursing internship; ^4^Frequency and percentage add up to more than 100% since multiple responses were possible; ^5^Students are required to complete a 90-day internship in Nursing in the first two years of their medical education program, ^6^Students are required to complete at least four clinical electives of 30 days each, ^7^Students are required to complete one clinical internship year at the end of their medical education; ^8^Frequencies and percentages not adding up to the total number of participants indicate “no response” entries; ^9^Items are ordered from highest to lowest frequency; ^10^No medical faculty in Bremen

### Attitudes toward the provision of consultation recordings

**Fig. 1:**
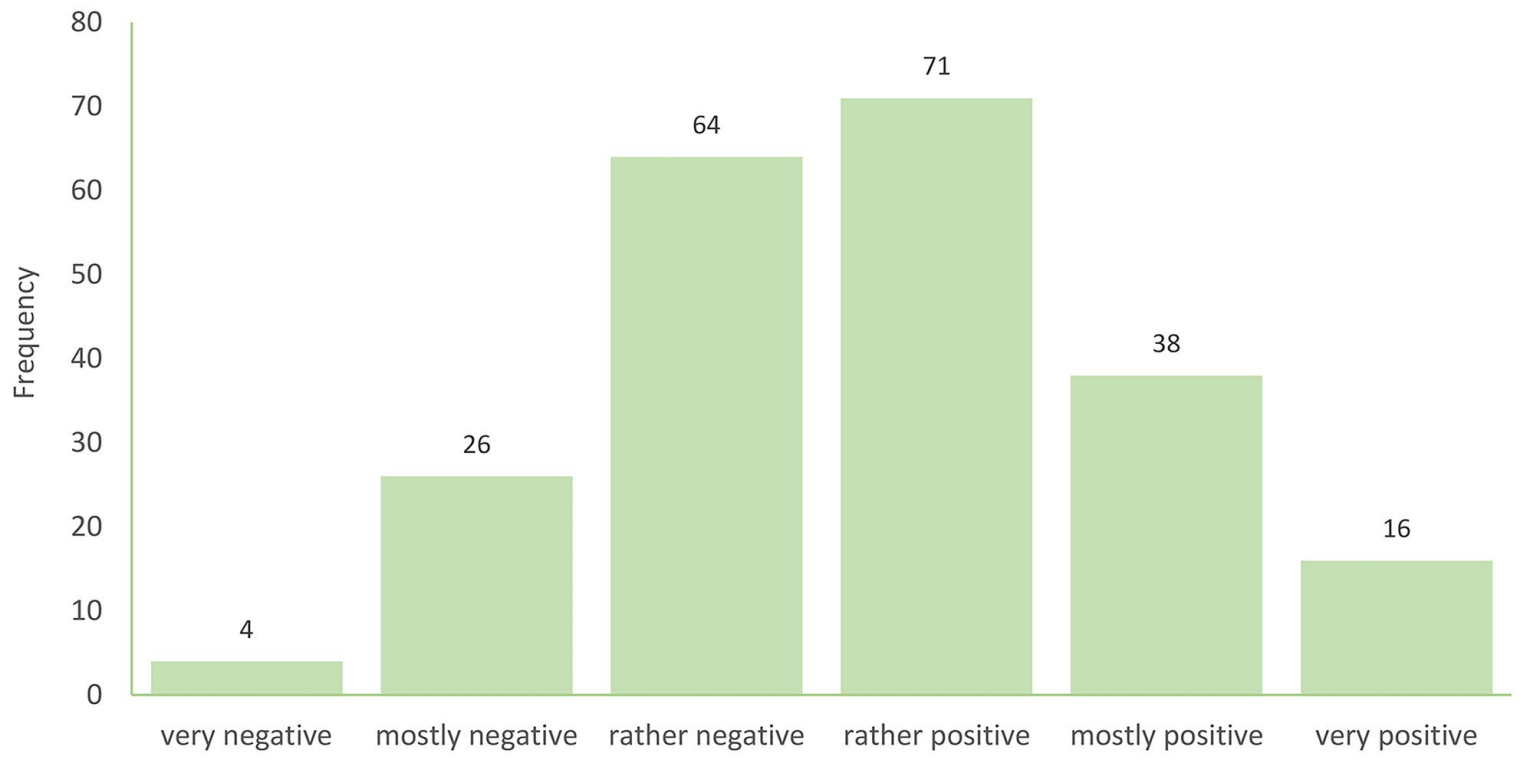
Attitude toward the provision of audio recordings of medical encounters for patients

Participants’ general attitude toward consultation recordings, measured on a scale from *very negative* (= 1) to *very positive* (= 6)), had a mean value of 3.74 (SD = 1.16, Median = 4.00, IQR = 3.00–4.00). Approximately half of the participants (n = 125, 56.3%) reported a *rather positive*, *mostly positive*, or *very positive* attitude toward consultation recordings (see Fig. 1). The item ”I would allow patients to make a consultation recording, but I would not proactively offer it” had a mean value of 3.95 (SD = 1.36, Median = 4.00, IQR = 3.00–5.00) on a scale from *does not apply* (= 1) to *fully applies* (= 6). The item ”In principle, it would be okay for me to offer a consultation recording to patients” had a mean value of 4.06 (SD = 1.46, Median = 4.00, IQR = 3.00–5.00) on the same scale.

Table 2 displays various statements regarding the potential benefits of consultation recordings and participants’ level of agreement, rated on a scale from *completely disagree* (= 1) to *completely agree* (= 6), ranked by mean score from highest to lowest. Supplementary File 3 includes frequency distribution graphs for all statements about potential benefits. Overall, participants expressed moderate to very high agreement with the proposed benefits of consultation recordings (means from 2.72 to 5.18, medians from 3.00 to 6.00). The highest levels of agreement were for the following statements: Consultation recordings could improve recall of the information discussed (X̅=5.18; SD = 1.04; Median = 6.00, IQR = 5.00–6.00), enhance preparation for follow-up appointments (X̅=4.75; SD = 1.18; Median = 5.00, IQR = 5.00–6.00), and improve understanding of information (X̅=4.61; SD = 1.19; Median = 5.00, IQR = 4.00–6.00). Furthermore, consultation recordings could provide evidence of what was said and done (X̅=4.58; SD = 1.13; Median = 5.00, IQR = 4.00–6.00) and allow patients to verify correct understanding of information (X̅=4.55; SD = 1.21; Median = 5.00, IQR = 4.00–6.00).

**Table 2:** Levels of agreement toward different statements about benefits of consultation recordings^1^

A consultation recording…^2^	*n* ^3^	Mean (SD)^#^	Median (IQR)^*^
…allows patients to have a better recall of the information discussed.	*222*	*5.18 (1.04)*	*6.00 (5.00–6.00)* ^*^
…allows patients to prepare for follow-up appointments (e.g. note down questions).	*222*	*4*,*75 (1.18)*	*5.00 (5.00–6.00)* ^*^
…enhances the understanding of information.	*220*	*4.61 (1.19)*	*5.00 (4.00–6.00)* ^*^
…provides evidence of what was said and done.	*219*	*4.58 (1.13)*	*5.00 (4.00–6.00)* ^*`^
…allows patients to retrospectively verify correct understanding of the information.	*222*	*4.55 (1.21)*	*5.00 (4.00–6.00)* ^*^
…allows patients to share information with their relatives.	*221*	*4.51 (1.24)*	*5.00 (4.00–5.00)* ^*^
…allows a better adherence to medical instructions.	*222*	*4.32 (1.18)* ^*#*^	*4.00 (4.00–5.00)* ^*^
…provides evidence in case of malpractice.	*215*	*4.31 (1.30)* ^*#*^	*4.00 (4.00–5.00)*
…is especially helpful in consultations in which treatment decisions are made.	*218*	*4.28 (1.28)* ^*#*^	*4.00 (4.00–5.00)*
…is especially helpful in complex and lengthy treatments.	*217*	*4.26 (1.36)*	*4.00 (4.00–5.00)* ^*^
…is especially helpful when starting or changing a treatment.	*214*	*4.19 (1.21)* ^*#*^	*4.00 (4.00–5.00)*
…is especially helpful for people with language barriers.	*220*	*4.16 (1.37)*	*4.00 (3.00–5.00)* ^*^
…allows relatives to provide better support to the patient.	*220*	*4.07 (1.18)* ^*#*^	*4.00 (3.00–5.00)*
…allows patients to compare their treatment options and make the best decision.	*219*	*4.06 (1.08)* ^*#*^	*4.00 (3.00–5.00)*
…is especially helpful for people with cognitive deficits.	*218*	*3.98 (1.42)* ^*#*^	*4.00 (3.00–5.00)*
…allows patients to ensure that the physician has understood them correctly.	*222*	*3.97 (1.18)*	*4.00 (3.00–5.00)* ^*^
…encourages patients to engage with their diagnosis.	*218*	*3.90 (1.12)* ^*#*^	*4.00 (3.00–5.00)*
…is especially helpful for older people.	*218*	*3.89 (1.34)* ^*#*^	*4.00 (3.00–5.00)*
…allows patients to share information with other healthcare professionals.	*219*	*3.74 (1.49)*	*4.00 (3.00–5.00)* ^*^
…should also be conducted when the diagnosis is communicated during the consultation.	*212*	*3.62 (1.50)*	*4.00 (3.00–5.00)* ^*^
…is helpful for treatment planning.	*217*	*3.54 (1.30)*	*3.00 (3.00–4.00)* ^*^
…facilitates patients’ active and self-responsible managing of their disease.	*213*	*3.53 (1.04)* ^*#*^	*4.00 (3.00–4.00)*
…facilitates an equal collaboration between patient and physician.	*212*	*3.49 (1.14)* ^*#*^	*3.00 (3.00–4.00)*
…allows physicians to be more responsive of concerns and needs of patients.	*212*	*3.48 (1.15)* ^*#*^	*3.50 (3.00–4.00)*
…provides protection for patients and physicians.	*215*	*3.46 (1.36)* ^*#*^	*3.00 (3.00–4.00)*
…improves the quality of communication.	*215*	*3.29 (1.27)*	*3.00 (3.00–4.00)* ^*^
…improves the trust between patients and physicians.	*210*	*2.99 (1.05)*	*3.00 (2.00–4.00)* ^*^
… reduces consultation length.	*213*	*2.73 (1.12)*	*3.00 (2.00–3.00)* ^*^
…should be made even in brief consultations with little amount of new information.	*218*	*2.72 (1.31)*	*3.00 (2.00–3.00)* ^*^

Notes. ^#^items are approximately normally distributed ^*^items are not normally distributed ^1^answers were assessed on a 6-point Likert scale, ranging from *completely disagree (= 1)* to *completely agree (= 6);*^2^Items are ordered from highest to lowest mean; ^3^Sample sizes (n) not adding up to the total number of participants indicate “no response” entries

Participants’ concerns regarding consultation recordings, also rated on a scale from *completely disagree* (= 1) to *completely agree* (= 6), ranged from moderate to very high agreement with the proposed statements (means from 2.88 to 5.30, medians from 3.00 to 6.00; see Table 3 and Supplementary File 4). The primary concerns centered on the misuse of consultation recordings, for example that the recordings could be shared without permission (X̅=5.30; SD = 1.06; Median = 6.00, IQR = 5.00–6.00), that physicians could feel pressured (X̅=5.24; SD = 1.01; Median = 6.00, IQR = 5.00–6.00), and about confidentiality and data protection (X̅=5.21; SD = 1.15; Median = 6.00, IQR = 5.00–6.00). In addition, they were concerned about what happens with the consultation recording (X̅=5.14; SD = 1.12; Median = 6.00, IQR = 5.00–6.00) and that it could be used as evidence against physicians (X̅=5.05; SD = 1.23; Median = 6.00, IQR = 4.00–6.00).

**Table 3:** Levels of agreement toward different statements about concerns regarding consultation recordings

I am concerned… ^1^	*n* ^3^	Mean (SD)	Median (IQR)
… that consultation recordings could be passed on undesirably.	*222*	*5.30 (1.06)*	*6.00 (5.00–6.00)* ^*^
…that a consultation recording would put pressure on physicians.	*221*	*5.24 (1.01)*	*6.00 (5.00–6.00)* ^*^
…about confidentiality and data protection if consultations were recorded.	*221*	*5.21 (1.15)*	*6.00 (5.00–6.00)* ^*^
…about what happens with the consultation recording.	*222*	*5.14 (1.12)*	*6.00 (5.00–6.00)* ^*^
…that a consultation recording would be used as evidence against physicians.	*221*	*5.05 (1.23)*	*6.00 (4.00–6.00)* ^*^
…that relatives could pressure patients into allowing them to listen to their consultation recording.	*220*	*4.96 (1.09)*	*5.00 (4.00–6.00)* ^*^
… that patients overinterpret statements made by the physician on the recording.	*220*	*4.90 (1.03)*	*5.00 (4.00–6.00)* ^*^
…that physicians would be reserved and less open if consultations were recorded.	*222*	*4.88 (1.15)*	*5.00 (4.00–6.00)* ^*^
…that the recording of the consultation could be distorted.	*220*	*4.62 (1.53)*	*5.00 (3.00–6.00)* ^*^
…that the physician-patient-relationship would be more formal if consultations were recorded.	*220*	*4.51 (1.23)*	*5.00 (4.00-5.75)* ^*^
…about patients perceiving a recording device as stressful during consultations.	*222*	*4.40 (1.21)*	*4.00 (4.00–5.00)* ^*^
…that patients would be reserved and less open if consultations were recorded.	*222*	*3.97 (1.33)* ^*#*^	*4.00 (3.00–5.00)*
…that the trust between patients and physicians would decrease if consultations were recorded.	*206*	*3.89 (1.21)* ^*#*^	*4.00 (3.00–5.00)*
…that consultation recordings prolong consultations.	*214*	*3.89 (1.29)*	*4.00 (3.00–5.00)* ^*^
…that a consultation recording disrupts clinical routines.	*214*	*3.63 (1.36)* ^*#*^	*4.00 (3.00–5.00)*
…that the quality of the communication decreases through a consultation recording.	*213*	*3.60 (1.29)*	*4.00 (3.00–4.00)* ^*^
…that the technical requirements for making consultation recordings do not exist.	*216*	*3.47 (1.64)*	*3.00 (2.00–5.00)* ^*^
…that listening to the consultation recording would be a psychological burden for patients.	*217*	*3.41 (1.26)* ^*#*^	*3.00 (3.00–4.00)*
…that recording consultations is too complicated for patients.	*218*	*3.29 (1.40)* ^*#*^	*3.00 (2.00–4.00)*
…that recording consultations is too complicated for physicians.	*219*	*3.17 (1.43)*	*3.00 (2.00–4.00)* ^*^
…that a consultation recording puts too much responsibility on patients.	*221*	*2.88 (1.16)*	*3.00 (2.00–3.00)* ^*^

Notes. ^#^items are approximately normally distributed ^*^items are not normally distributed ^1^answers were assessed on a 6-point Likert scale, ranging from *completely disagree (= 1)* to *completely agree (= 6);*^2^Items are ordered from highest to lowest agreement; ^3^Sample sizes (n) not adding up to the total number of participants indicate “no response” entries

### Association between gender and regular patient contact with attitudes toward consultation recordings

Descriptively, the median attitude score for both groups was 4.00, with IQR [3.00–4.00] for female and [3.00–5.00] for male participants. There was no statistically significant difference in attitudes between female (MRank = 109.74) and male (MRank = 105.50) participants (U = 4630.500, Z = -0.469, *p* =.641).

Regarding the groups having regular patient contact or not, the median attitude score for both groups was 4.00, with IQR [3.00–5.00] for participants with regular patient contact and [3.00–4.00] for participants with no regular patient contact. There was no statistically significant difference in median attitudes toward consultation recordings between medical students with regular patient contact and medical students with no regular patient contact (U = 5945.500, Z = -0.107, *p* =.915).

### Desire for future consultation recordings

Participants were asked whether they would like to offer consultation recordings to patients in the future. Of the 222 participants, 35 (15.8%) answered “yes”, 89 (40.1%) answered “maybe”, and 98 (44.1%) answered “no”. Among those who responded “yes” or “maybe” (*n* = 124), 48 (38.7%) indicated they would be open to patients recording consultations with their own cell phone, while 76 (61.3%) would not. Regarding access to the recordings, 122 of those 124 participants (98.4%) thought that they should have access to the recording themselves, while 2 (1.6%) did not. When asked if they would listen to the recording afterward, 24 of 124 participants (19.4%) said that they would not, 33 (26.6%) said they would, and 67 (54.0%) reported they would listen only in specific situations, such as misunderstandings, conflicts, or for their own reassurance.

All participants were asked whether they would consider using consultation recordings if they or a relative were the patient. Seventy-eight participants (35.1%) answered “yes”, 67 (30.2%) answered “maybe”, and 77 (34.7%) answered “no”.

## Discussion

This first investigation into medical students’ attitudes toward providing patients with audio recordings of their medical encounters revealed mixed views. While over half of the participants expressed generally positive attitudes and recognized the potential benefits of consultation recordings, they also strongly endorsed concerns and risks associated with this intervention. Our sample of medical students showed attitudes toward consultation recordings that were similar to those observed in previous studies with physicians [10, 16, 26, 27, 30]. Overall, our sample of students was slightly more positive, particularly regarding the perceived benefits. Participating students emphasized the informational benefits for patients, such as improved recall and understanding of medical information and the value of recordings as evidence and for sharing information with relatives, which mirror fundamental aspects of patient-centered care [44]. Additionally, our results suggest that neither gender nor regular patient contact significantly influence attitudes toward consultation recordings in this sample. Over half of the participants expressed openness to offering consultation recordings to patients in the future, more than the oncologists in the previous German study, but only a small proportion demonstrated a definite willingness to do so.

Participants’ concerns centered on potential mishandling of the recordings, legal implications, and feeling pressured by the practice. The high levels of agreement on concerns related to consultation recordings could be associated with the limited clinical experience of medical students, in other words, no practical experience with providing consultation recordings to patients. This hypothesis is supported by study results indicating older physicians with more clinical experience are more favorable toward consultation recordings [26]. Conversely, this limited clinical experience may also contribute to greater openness among medical students toward consultation recordings, as they may not yet fully share the medico-legal and practical concerns that practicing physicians face. On the other hand, a recent meta-analysis revealed a relatively negative attitude toward patient-centered care in medical students [38], which can be a contributing factor to the hesitancy toward this patient-centered intervention. Furthermore, physicians and patients in Germany reported limited experience with this intervention compared with other countries [10, 16, 26, 30]. This low prevalence of consultation recordings in Germany might have an influence on the apprehensiveness of medical students as well. Still, participants agreed that consultation recordings could be beneficial for patients. These findings are consistent with results from international research [2, 10, 16, 22, 24–27].

Previous research shows that physicians worry about losing control over the consultation process [16, 24, 26, 30], a concern also evident among our student sample. Their openness to consultation recordings, provided they maintain access to the recordings, underscores the need for measures that ensure transparency and protect both patient and physician interests [16, 24–26, 32, 45]. Research suggest that proactively offering patients the option to record consultations fosters trust and openness, while helping physicians to maintain control [18, 32, 46, 47]. In addition, this can circumvent covert recordings [18, 46, 47]. Another proposed solution is the implementation of provider-led recordings made available through healthcare services, such as a smartphone app, which also ensures that a copy of the recording is preserved in the hospital’s medical record [19, 29]. These approaches could also help future physicians in Germany to adapt to the increasing use of digital technologies in healthcare [48–50], a trend that is still less prevalent compared to other countries [10, 16, 18, 26]. The reported hesitancy toward consultation recordings may stem from a general skepticism toward digital innovations and shifts in power dynamics [48]. Germany’s dual party consent law may also play a role in the reluctance to adopt interventions like consultation recordings, as it requires both parties to consent before a recording can be made, unlike the one-party consent laws in most US states and in the UK [51].

The mixed attitudes of (future) physicians highlight a potential barrier to implementing consultation recordings in healthcare delivery in Germany. This is in line with previous research suggesting that physician hesitancy and skepticism about consultation recordings’ benefits hinders implementation [11, 16, 25]. Providing opportunities for positive experiences and information about the evidence-based benefits of consultation recordings during medical and postgraduate education as well as in clinical practice could help foster acceptance [11, 16, 25]. While limited clinical experience may or may not be associated with more positive attitudes toward consultation recordings, we assume that increased positive experience with consultation recordings would lead to increased acceptance. This has also been found in prior studies [11, 16, 25, 26]. Integrating consultation recordings into medical school curricula, particularly within courses on patient-centered care or digital health innovations, while strengthening education on the concept of patient-centered care [52], could further support this effort. Additionally, the perception and approach of role models toward patient-centeredness play a crucial role in medical education [53–55]. Therefore, having physicians who demonstrate a positive approach to patient-centered interventions, such as consultation recordings, can significantly contribute to their future adoption. In recent years, the German medical curricula have made significant strides in incorporating communication skills, patient-centered care, and shared decision-making [56]. Many medical faculties have successfully included shared decision making as a mandatory component [56]. Some curricula include specialized lectures and seminars featuring role-playing with simulated patients to enhance practical skills [56]. Further research should focus on the integration of patient-centered interventions in the education of medical students as well as in the training of physicians. However, the physicians’ hesitancy regarding consultation recordings could also hinder them teaching about consultation recordings. To address the concerns toward consultation recordings, pilot testing the intervention in routine care could further allow positive experiences for physicians. In turn, this could foster medical education on consultation recordings. Additionally, subsequent studies should adopt more robust study designs that go beyond descriptive analyses.

A key strength of our study is that it is one of the first to explore consultation recordings in Germany and, to our knowledge, the first to examine medical students’ attitudes, thus addressing an important research gap. The quantitative questionnaire based on a thoroughly developed questionnaire is another strength. A limitation of this study is a potential selection bias (participating medical students might have had a more favorable attitude toward consultation recordings than non-participants), which limits the generalizability of our results. Furthermore, most participants in our sample were in their first years of medical education and had limited clinical experience in general and thus expressed their attitudes on an intervention that they had no experience with. Additional limitations include the lack of investigation into reasons for non-participation and having no standardized psychometrically tested questionnaire to assess attitudes toward consultation recordings.

This study reveals mixed attitudes among medical students in Germany toward consultation recordings, highlighting both recognition of their patient-centered benefits as well as concerns about risks such as legal implications and loss of control. Limited clinical experience and the low prevalence of consultation recordings in Germany likely contribute to these apprehensions. Integrating patient-centered interventions such as consultation recordings into medical education and providing role models who support patient-centered care could foster greater acceptance. Addressing legal and practical concerns may further promote their adoption. Overall, consultation recordings have the potential to become a valuable tool in routine healthcare if current barriers are effectively addressed.

## Electronic supplementary material

Below is the link to the electronic supplementary material.

Supplementary Material 1

Supplementary Material 2

Supplementary Material 3

Supplementary Material 4

## Data Availability

The data supporting the findings of this study are available from the corresponding author on reasonable request.

## Declarations

### Declaration of AI and AI-assisted technologies in the writing process

During the preparation of this work the authors used the software ChatGPT to improve readability and language. After using this tool, the authors reviewed and edited the content as needed and take full responsibility for the content of the publication.
