# Supplementary material for: Medical students’ attitudes toward providing patients with audio recordings of their medical encounters: a cross-sectional online survey
Source: BMC Med Educ. 2025 Jun 19;25:853. doi: 10.1186/s12909-025-07460-9 (PMC12180275; doi:10.1186/s12909-025-07460-9)
Supplement: Supplementary file 2 — Supplementary Material 2 [file 12909_2025_7460_MOESM2_ESM.docx]

**SUPPLEMENTARY FILE 2**

**Quantitative online survey**
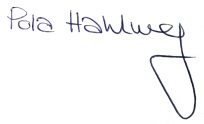

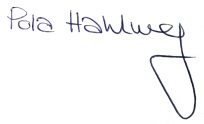

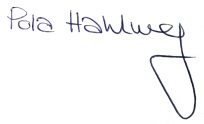

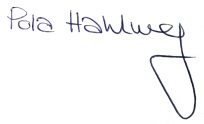

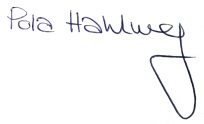


| Today’s date | | | | | |  |  |  |  |  |  |
| --- | --- | --- | --- | --- | --- | --- | --- | --- | --- | --- | --- |
|  |  |  |  |  |  | D | D | M | M | Y | Y |

| **The provision of audio recordings of medical encounters for patients**  Information and Consent Form for Participation in an Online Survey.  Dear Student,  We would like to understand your thoughts on providing audio recordings of their own medical encounters to patients with cancer. We are particularly interested in the perspectives of medical students. Your participation is crucial to this project.  We kindly ask you to complete the following anonymous questionnaire. It includes questions about your attitudes, as well as some questions about yourself (e.g., age, gender, education, professional experience).  Filling out the questionnaire will take approximately 10-15 minutes. As a token of appreciation for your participation, you can take part in a raffle for 25 vouchers worth 10 euros each at the end of the survey. The personal information required for this (e-mail address) will be stored separately from your questionnaire and cannot be linked to your responses. Providing this information is voluntary.  Your participation is voluntary. Your data will only be used if you consent to participate. If you do not wish to participate, you will not face any disadvantages. You can withdraw at any time, even after giving consent and without providing reasons, by simply closing the window.  For the sake of readability, in some places, we have omitted the simultaneous use of male, female, and diverse gender forms (m/w/d). All references to individuals apply equally to all genders.  **Project team:**  Marina Buzo (Master’s Student), Prof. Dr. Isabelle Scholl (Principal Investigator), Cheyenne Topf (Research Associate)  If you have read and understood the project and data protection information and wish to participate in the survey, please consent below. Please save this PDF so you can refer to your consent at any time. If you do not wish to participate, you do not need to take any action. |
| --- |

|  | **Declaration of Consent**  Under the conditions mentioned above and in the PDF, I consent to participate in the research project described above and agree that my data may be processed and used in accordance with the preceding information. | |
| --- | --- | --- |
|  | 🞎 Yes | 🞎 No |

| 1. | **Are you currently enrolled at a German university to study human medicine?** | |
| --- | --- | --- |
|  | 🞎 Yes | 🞎 No |

| **Attitudes towards the provision of audio recordings of medical encounters**  The following questions concern your attitudes towards providing audio recordings of medical encounters. Please answer based on your current feelings. There are no right or wrong answers. Please try to provide your level of agreement spontaneously and generally. **Please answer all questions, even if you have not yet conducted a doctor-patient consultation yourself.** | | | | | | | | | | | | | |
| --- | --- | --- | --- | --- | --- | --- | --- | --- | --- | --- | --- | --- | --- |
|  | **In principle, it would be okay for me to offer a consultation recording to patients.** | | | | | | | | | | | | |
|  | 🞎 Does not apply at all | 🞎 Mostly does not apply | 🞎 Somewhat does not apply | | 🞎 Somewhat applies | | 🞎 Mostly applies | | 🞎 Fully applies | | | 🞎No response | |
|  | **I would allow patients to make a consultation recording, but I would not proactively offer it.** | | | | | | | | | | | | |
|  | 🞎 Does not apply at all | 🞎 Mostly does not apply | 🞎 Somewhat does not apply | | 🞎 Somewhat applies | | 🞎 Mostly applies | | 🞎 Fully applies | | | 🞎No response | |
| 4 | **How would you describe your attitude towards the provision of consultation recordings for patients?** | | | | | | | | | | | | |
|  | 🞎 Very negative | 🞎 Mostly negative | 🞎 Somewhat negative | | 🞎 Somewhat positive | | 🞎 Mostly positive | | 🞎 Very positive | | | 🞎No response | |
| **Please check the extent to which you agree with the following statements. When the term „consultation recording“ is used, it refers to the audio recording of medical encounters.** | | | | | | | | | | | | | |
|  |  | | | Completely disagree | | Partially disagree | Slightly disagree | Slightly agree | | Partially agree | Completely agree | | No Response |
| 5 | A consultation recording enhances the understanding of information. | | | 🞎 | | 🞎 | 🞎 | 🞎 | | 🞎 | 🞎 | | 🞎 |
| 6 | A consultation recording allows patients to retrospectively verify correct understanding of the information. | | | 🞎 | | 🞎 | 🞎 | 🞎 | | 🞎 | 🞎 | | 🞎 |
|  | A consultation recording allows patients to ensure that the physician has understood them correctly. | | | 🞎 | | 🞎 | 🞎 | 🞎 | | 🞎 | 🞎 | | 🞎 |
|  | A consultation recording allows patients to have a better recall of the information discussed. | | | 🞎 | | 🞎 | 🞎 | 🞎 | | 🞎 | 🞎 | | 🞎 |
|  | A consultation recording allows patients to prepare for follow-up appointments (.e.g. note down questions) | | | 🞎 | | 🞎 | 🞎 | 🞎 | | 🞎 | 🞎 | | 🞎 |
|  | A consultation recording improves the quality of communication. | | | 🞎 | | 🞎 | 🞎 | 🞎 | | 🞎 | 🞎 | | 🞎 |
|  | I am concerned that the quality of the communication decreases through a consultation recording. | | | 🞎 | | 🞎 | 🞎 | 🞎 | | 🞎 | 🞎 | | 🞎 |
|  | A consultation recording allows physicians to be more responsive of concerns and needs of patients. | | | 🞎 | | 🞎 | 🞎 | 🞎 | | 🞎 | 🞎 | | 🞎 |
|  | I am concerned that patients would be reserved and less open if consultations were recorded. | | | 🞎 | | 🞎 | 🞎 | 🞎 | | 🞎 | 🞎 | | 🞎 |
|  | I am concerned that physicians would be reserved and less open if consultations were recorded. | | | 🞎 | | 🞎 | 🞎 | 🞎 | | 🞎 | 🞎 | | 🞎 |
|  | I am concerned that patients over-interpret statements made by the physician on the recording. | | | 🞎 | | 🞎 | 🞎 | 🞎 | | 🞎 | 🞎 | | 🞎 |
|  | A consultation recording improves the trust between patients and physicians. | | | 🞎 | | 🞎 | 🞎 | 🞎 | | 🞎 | 🞎 | | 🞎 |
|  | I am concerned that the trust between patients and physicians would decrease if consultations were recorded. | | | 🞎 | | 🞎 | 🞎 | 🞎 | | 🞎 | 🞎 | | 🞎 |
|  | I am concerned that the physician-patient-relationship would be more formal if consultations were recorded. | | | 🞎 | | 🞎 | 🞎 | 🞎 | | 🞎 | 🞎 | | 🞎 |
|  | I am concerned that a consultation recording would put pressure on physicians. | | | 🞎 | | 🞎 | 🞎 | 🞎 | | 🞎 | 🞎 | | 🞎 |
|  | A consultation recording facilitates an equal collaboration between patient and physician. | | | 🞎 | | 🞎 | 🞎 | 🞎 | | 🞎 | 🞎 | | 🞎 |
|  | A consultation recording facilitates patients’ active and self-responsible managing of their disease. | | | 🞎 | | 🞎 | 🞎 | 🞎 | | 🞎 | 🞎 | | 🞎 |
|  | A consultation recording allows patients to compare their treatment options and make the best decision. | | | 🞎 | | 🞎 | 🞎 | 🞎 | | 🞎 | 🞎 | | 🞎 |
|  | A consultation recording encourages patients to engage with their diagnosis. | | | 🞎 | | 🞎 | 🞎 | 🞎 | | 🞎 | 🞎 | | 🞎 |
|  | I am concerned that a consultation recording puts too much responsibility on patients. | | | 🞎 | | 🞎 | 🞎 | 🞎 | | 🞎 | 🞎 | | 🞎 |
|  | I am concerned that listening to the consultation recording would be a psychological burden for patients. | | | 🞎 | | 🞎 | 🞎 | 🞎 | | 🞎 | 🞎 | | 🞎 |
|  | A consultation recording allows patients to share information with other health care professionals. | | | 🞎 | | 🞎 | 🞎 | 🞎 | | 🞎 | 🞎 | | 🞎 |
|  | A consultation recording allows patients to share information with their relatives. | | | 🞎 | | 🞎 | 🞎 | 🞎 | | 🞎 | 🞎 | | 🞎 |
|  | A consultation recording allows relatives to provide better support to the patient. | | | 🞎 | | 🞎 | 🞎 | 🞎 | | 🞎 | 🞎 | | 🞎 |
|  | I am concerned that relatives could pressure patients into allowing them to listen to their consultation recording. | | | 🞎 | | 🞎 | 🞎 | 🞎 | | 🞎 | 🞎 | | 🞎 |
|  | A consultation recording provides evidence of what was said and done. | | | 🞎 | | 🞎 | 🞎 | 🞎 | | 🞎 | 🞎 | | 🞎 |
|  | A consultation recording provides a protection for patients and physicians. | | | 🞎 | | 🞎 | 🞎 | 🞎 | | 🞎 | 🞎 | | 🞎 |
|  | A consultation recording provides evidence in case of malpractice. | | | 🞎 | | 🞎 | 🞎 | 🞎 | | 🞎 | 🞎 | | 🞎 |
|  | I am concerned that a consultation recording would be used as evidence against physicians. | | | 🞎 | | 🞎 | 🞎 | 🞎 | | 🞎 | 🞎 | | 🞎 |
|  | I am concerned what happens with the consultation recording. | | | 🞎 | | 🞎 | 🞎 | 🞎 | | 🞎 | 🞎 | | 🞎 |
|  | I am concerned about confidentiality and data protection if consultations were recorded. | | | 🞎 | | 🞎 | 🞎 | 🞎 | | 🞎 | 🞎 | | 🞎 |
|  | I am concerned that consultation recordings could be passed on undesirably. | | | 🞎 | | 🞎 | 🞎 | 🞎 | | 🞎 | 🞎 | | 🞎 |
|  | I am concerned that the recording of the consultation could be distorted. | | | 🞎 | | 🞎 | 🞎 | 🞎 | | 🞎 | 🞎 | | 🞎 |
|  | A consultation recording is helpful for treatment planning. | | | 🞎 | | 🞎 | 🞎 | 🞎 | | 🞎 | 🞎 | | 🞎 |
|  | A consultation recording allows a better adherence to medical instructions. | | | 🞎 | | 🞎 | 🞎 | 🞎 | | 🞎 | 🞎 | | 🞎 |
|  | I am concerned that a consultation recording disrupts clinical routines. | | | 🞎 | | 🞎 | 🞎 | 🞎 | | 🞎 | 🞎 | | 🞎 |
|  | I am concerned that the technical requirements for making consultation recordings don’t exist. | | | 🞎 | | 🞎 | 🞎 | 🞎 | | 🞎 | 🞎 | | 🞎 |
|  | I am concerned that recording consultations is too complicated for physicians. | | | 🞎 | | 🞎 | 🞎 | 🞎 | | 🞎 | 🞎 | | 🞎 |
|  | I am concerned that recording consultations is too complicated for patients. | | | 🞎 | | 🞎 | 🞎 | 🞎 | | 🞎 | 🞎 | | 🞎 |
|  | I am concerned about patients perceiving a recording device as stressful during consultations. | | | 🞎 | | 🞎 | 🞎 | 🞎 | | 🞎 | 🞎 | | 🞎 |
|  | I am concerned that consultation recordings prolong consultations. | | | 🞎 | | 🞎 | 🞎 | 🞎 | | 🞎 | 🞎 | | 🞎 |
|  | A consultation recording reduces consultation length. | | | 🞎 | | 🞎 | 🞎 | 🞎 | | 🞎 | 🞎 | | 🞎 |
|  | A consultation recording is especially helpful for older people. | | | 🞎 | | 🞎 | 🞎 | 🞎 | | 🞎 | 🞎 | | 🞎 |
|  | A consultation recording is especially helpful for people with language barriers. | | | 🞎 | | 🞎 | 🞎 | 🞎 | | 🞎 | 🞎 | | 🞎 |
|  | A consultation recording is especially helpful for people with cognitive deficits. | | | 🞎 | | 🞎 | 🞎 | 🞎 | | 🞎 | 🞎 | | 🞎 |
|  | A consultation recording is especially helpful when starting or changing a treatment. | | | 🞎 | | 🞎 | 🞎 | 🞎 | | 🞎 | 🞎 | | 🞎 |
|  | A consultation recording is especially helpful when treatments are complex and extensive. | | | 🞎 | | 🞎 | 🞎 | 🞎 | | 🞎 | 🞎 | | 🞎 |
|  | A consultation recording is especially helpful in consultations in which treatment decisions are made. | | | 🞎 | | 🞎 | 🞎 | 🞎 | | 🞎 | 🞎 | | 🞎 |
|  | A consultation recording should also be conducted when the diagnosis is communicated during the consultation. | | | 🞎 | | 🞎 | 🞎 | 🞎 | | 🞎 | 🞎 | | 🞎 |
|  | A consultation recording should be made even in brief consultations with little amount of new information. | | | 🞎 | | 🞎 | 🞎 | 🞎 | | 🞎 | 🞎 | | 🞎 |

| **Desire for the provision of audio recordings of medical encounters**  The following questions pertain to your potential preferences regarding the provision of audio recordings of your own medical encounters. | | | | |
| --- | --- | --- | --- | --- |
|  | **In the future, would you like to provide audio recordings of your medical consultations to your patients?** | | | |
|  | 🞎 Yes | 🞎 No | | 🞎 Maybe |
|  | *[If future desire „yes“ or „maybe“:]* **Should the audio recording also be available to you?** | | | |
|  | 🞎 Yes | | 🞎 No | |
|  | *[If future desire „yes“ or „maybe“:]* **Would you be willing to listen to such a recording after the consultation?** | | | |
|  | 🞎 Yes | 🞎 No | | 🞎 Yes, if…*(please specify):*  ______________________ |
|  | *[If future desire „yes“ or „maybe“:]* **Would you be open to patients making these consultation recordings on their cell phone?** | | | |
|  | 🞎 Yes | | 🞎 No | |
| 1. **1** | **Would you be interested in recording the consultation if you or a family member were the patient?** | | | |
|  | 🞎 Yes | 🞎 No | | 🞎 Maybe |

| **Your desire for participation** | | | | | |
| --- | --- | --- | --- | --- | --- |
|  | **Who, in your opinion, should make treatment decisions?**  Please select the statement that most closely aligns with your attitude. | | | | |
|  | Patients should make the decision what medical treatment they receive | Patients should ultimately make the decision about their medical treatment, after having seriously considered my medical opinion. | I prefer my patient and I to share the responsibility for making the decision which medical treatment is best for them. | I prefer to make the final decision about the patient's medical treatment, considering their opinion. | As a doctor, I prefer to make all decisions concerning the patient's medical treatment. |
|  | 🞎 | 🞎 | 🞎 | 🞎 | 🞎 |

| **Affinity for Technology Interaction** | | | | | | | | | | | | | | | | | | | | | | | | | | | | | | | |
| --- | --- | --- | --- | --- | --- | --- | --- | --- | --- | --- | --- | --- | --- | --- | --- | --- | --- | --- | --- | --- | --- | --- | --- | --- | --- | --- | --- | --- | --- | --- | --- |
| In the following questionnaire, we will ask you about your interaction with technical systems. The term “technical systems” refers to apps and other software applications, as well as entire digital devices (e.g., mobile phone, computer, TV, car navigation).  Please indicate the degree to which you agree/disagree with the following statements. | | | | | | | | | | | | | | | | | | | | | | | | | | | | | | | |
|  | |  | | | | | | | | | | Completely disagree | | Largely disagree | | | | | Slightly disagree | | | | Slightly agree | | | | Completely agree | | | Completely agree | |
|  | | I like to occupy myself in greater detail with technical systems. | | | | | | | | | | 🞎 | | 🞎 | | | | | 🞎 | | | | 🞎 | | | | 🞎 | | | 🞎 | |
|  | | I like testing the functions of new technical systems | | | | | | | | | | 🞎 | | 🞎 | | | | | 🞎 | | | | 🞎 | | | | 🞎 | | | 🞎 | |
|  | | It is enough for me that a technical system works; I don’t care how or why. | | | | | | | | | | 🞎 | | 🞎 | | | | | 🞎 | | | | 🞎 | | | | 🞎 | | | 🞎 | |
|  | | It is enough for me to know the basic functions of a technical system. | | | | | | | | | | 🞎 | | 🞎 | | | | | 🞎 | | | | 🞎 | | | | 🞎 | | | 🞎 | |
| **Knowledge about law** | | | | | | | | | | | | | | | | | | | | | | | | | | | | | | | |
|  | **Do you know the laws that regulate audio recordings of conversations in Germany?** | | | | | | | | | | | | | | | | | | | | | 🞎 Yes | | 🞎 No | | | 🞎 No response | | | | |
|  | **Please select the answer “yes”.** | | | | | | | | | | | | | | | | | | | | | 🞎 Yes | | 🞎 No | | | 🞎 Maybe | | | | 🞎 No response |
| **Questions about you**  Please provide some information about yourself below. | | | | | | | | | | | | | | | | | | | | | | | | | | | | | | | |
|  | | | **Age in years** | | | __________________________________________________________________________ | | | | | | | | | | | | | | | | | | | | | | | | | |
|  | | | **Gender** | | | 🞎 | Female | | | | 🞎 | | Male | | | | | 🞎 | | | Non binary/diverse | | | | | | |  |  | | |
|  | | | **Current semester** | | | __________________________________________________________________________ | | | | | | | | | | | | | | | | | | | | | | | | | |
|  | | | **Which medical state exams did you pass already? (multiple answers possible)** | | | | | | | | | | | | | | | | | | | | | | | | | | | | |
|  | | | 🞎 | First exam | | | | | 🞎 | Second exam | | | | | | | 🞎 | | | None yet | | | | | | | | | | | |
|  | | | **Which internships did you complete within the course of your university degree? (multiple answers possible)** | | | | | | | | | | | | | | | | | | | | | | | | | | | | |
|  | | | 🞎 | | Nursing internship | | | 🞎 | Clinical traineeship(s) (please specify number of months) __________ | | | | | | 🞎 | Clinical internship year | | | | | | | | | 🞎 | None yet | | | | | |
| 72 | | | **Do you have other training in a medical profession with direct patient contact (e.g., nursing, medical assistant)?** | | | | | | | | | | | | | | | | | | | | | | | | | | | | |
|  | | | 🞎 | | Yes | | | | | | | | | | 🞎 | No | | | | | | | | | | | | | | | |
| 73 | | | *[If „yes]* **How many years of work experience do you have working in this profession?** | | | | | | | | | | | | | | | | | | | | | | | | | | | | |
|  | | | 🞎 | | Less than 1 year | | | 🞎 | 1-3 years | | | | | | 🞎 | 4-6 years | | | | | | | | | 🞎 | More than 7 years | | | | | |
| 74 | | | **Do you have tasks with regular patient contact beyond your medical school education?** | | | | | | | | | | | | | | | | | | | | | | | | | | | | |
|  | | | 🞎 | | Yes | | | | | | | | | | 🞎 | No | | | | | | | | | | | | | | | |
|  | | | **In which federal state are you studying?** | | | | | | | | | | | | | | | | | | | | | | | | | | | | |
|  | | |  | 🞎 Schleswig-Holstein  🞎 Hamburg  🞎 Lower Saxony  🞎 Bremen  🞎 North Rhine-Westphalia  🞎 Hesse  🞎 Rhineland-Palatinate  🞎 Baden-Württemberg  🞎 Bavaria  🞎 Saarland  🞎 Berlin  🞎 Brandenburg  🞎 Mecklenburg-Western Pomerania  🞎 Saxony  🞎 Saxony-Anhalt  🞎 Thuringia | | | | | |  | | | | | | | | | | | | | | | | | | | | | |
