# Supplementary material for: Medical students’ attitudes toward providing patients with audio recordings of their medical encounters: a cross-sectional online survey
Source: BMC Med Educ. 2025 Jun 19;25:853. doi: 10.1186/s12909-025-07460-9 (PMC12180275; doi:10.1186/s12909-025-07460-9)
Supplement: Supplementary file 3 — Supplementary Material 3 [file 12909_2025_7460_MOESM3_ESM.docx]

**SUPPLEMENTARY FILE 3**

Frequency distributions of the attitudes toward and statements about benefits of consultation recordings, ranging from *completely disagree* *(=1)* to *completely agree (=6).*

*Ordered from highest to lowest mean.*
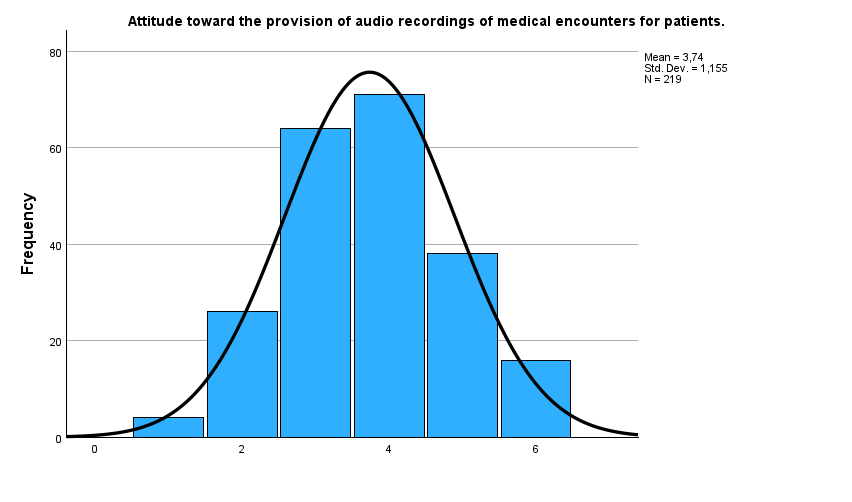

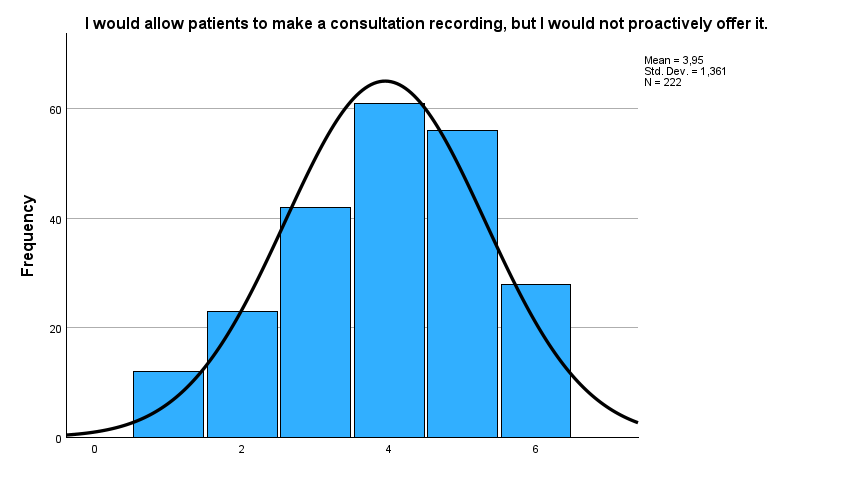

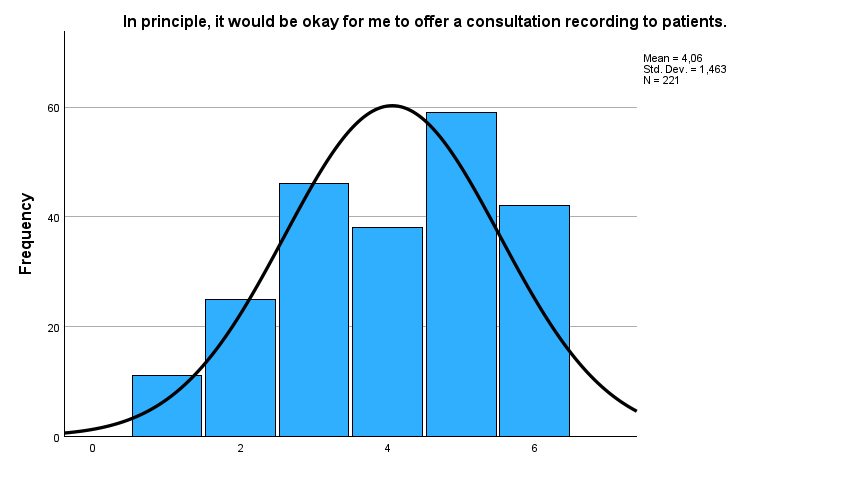


*
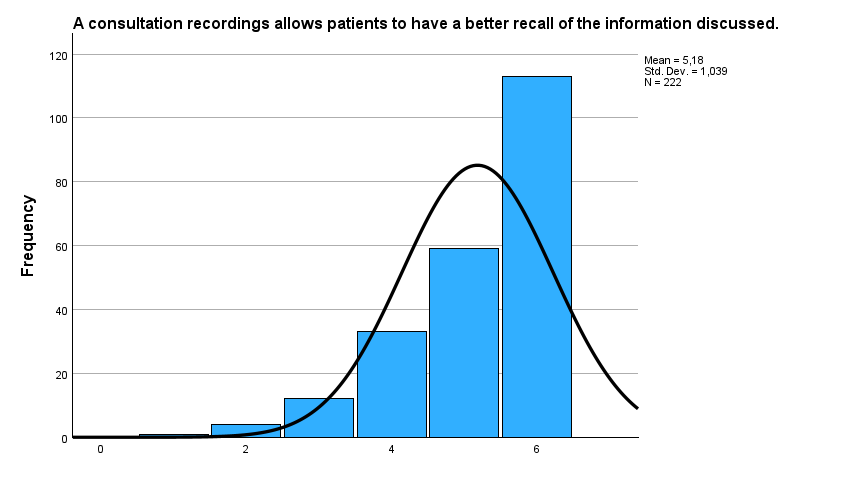

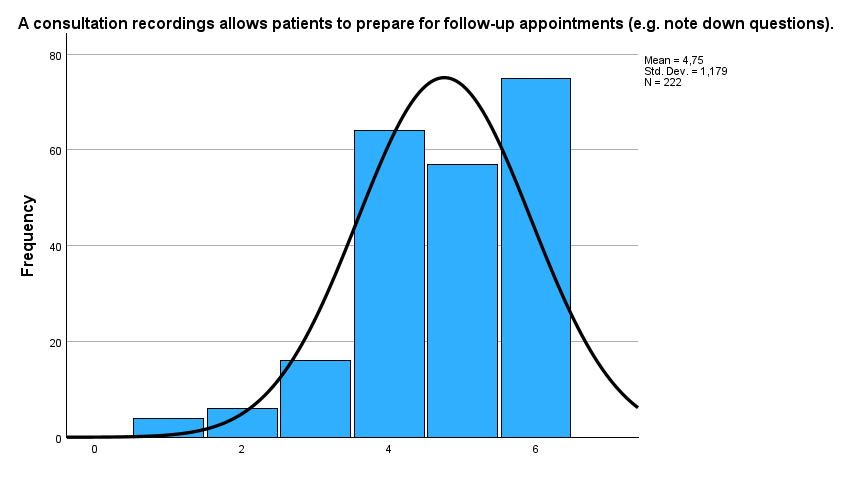
*

*
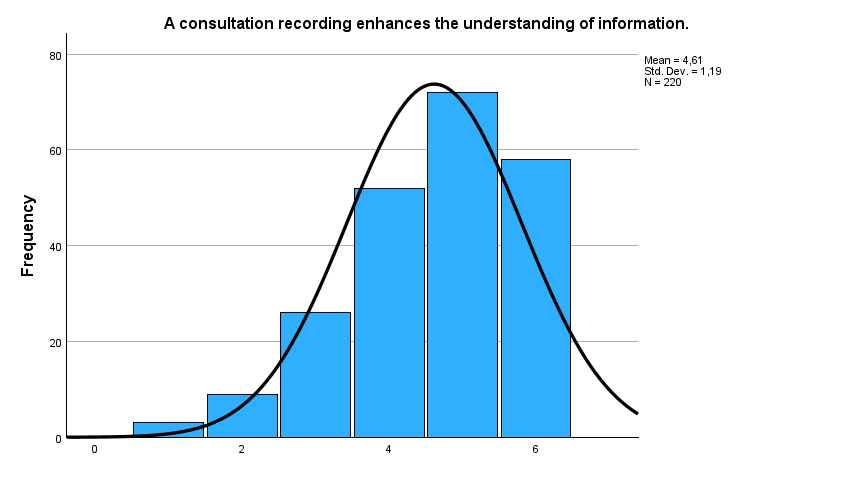

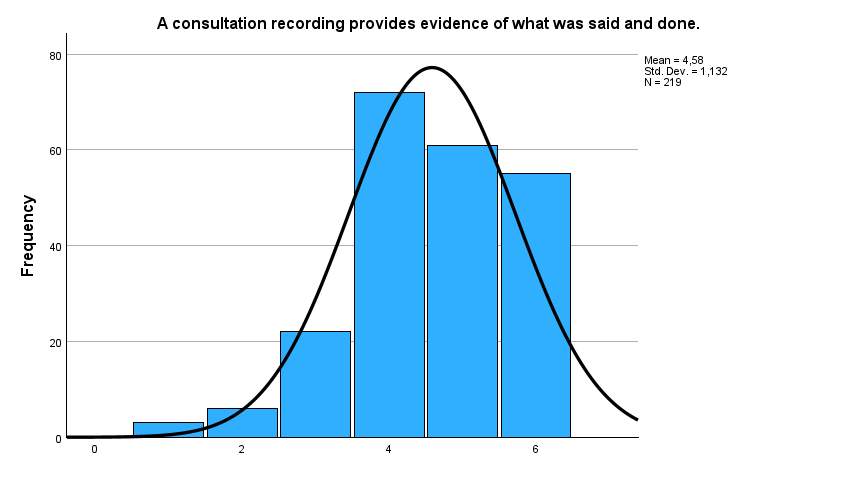
*

*
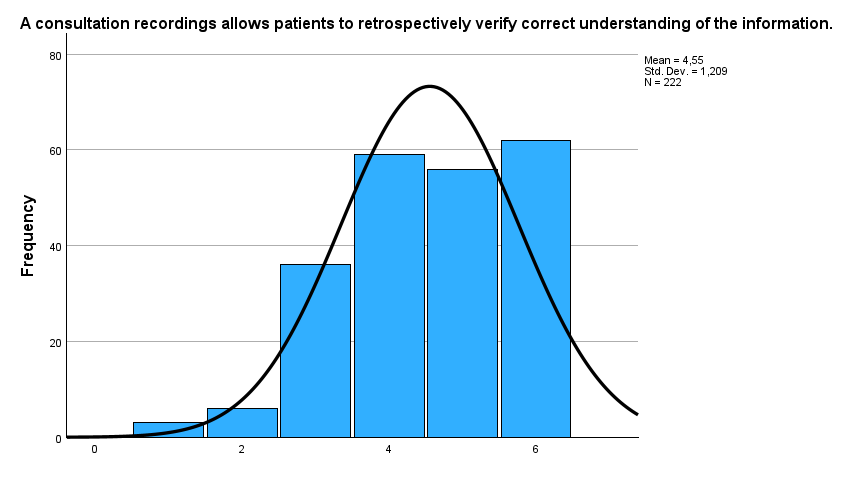

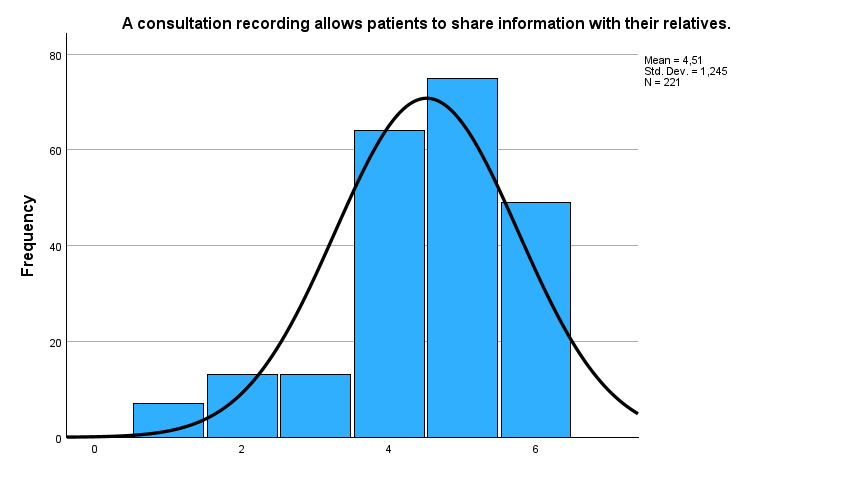

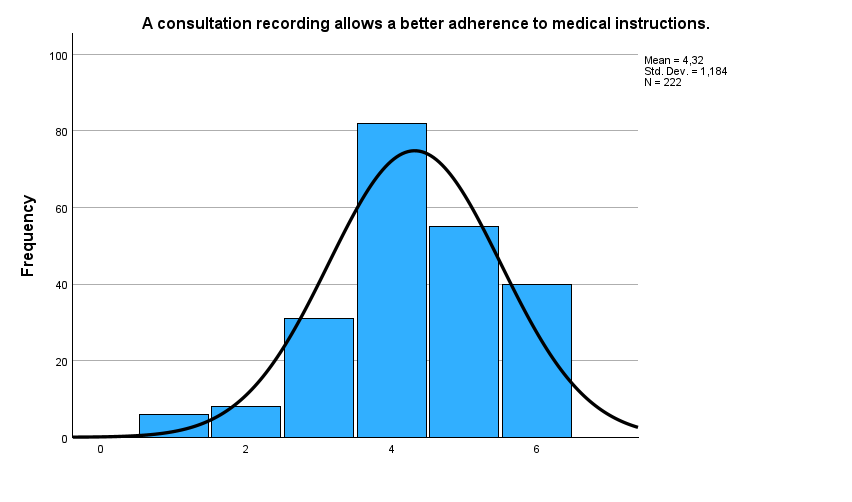

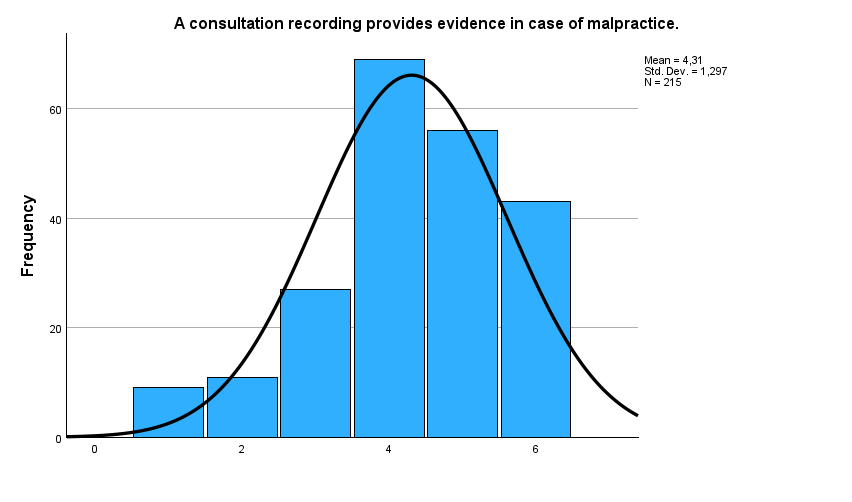

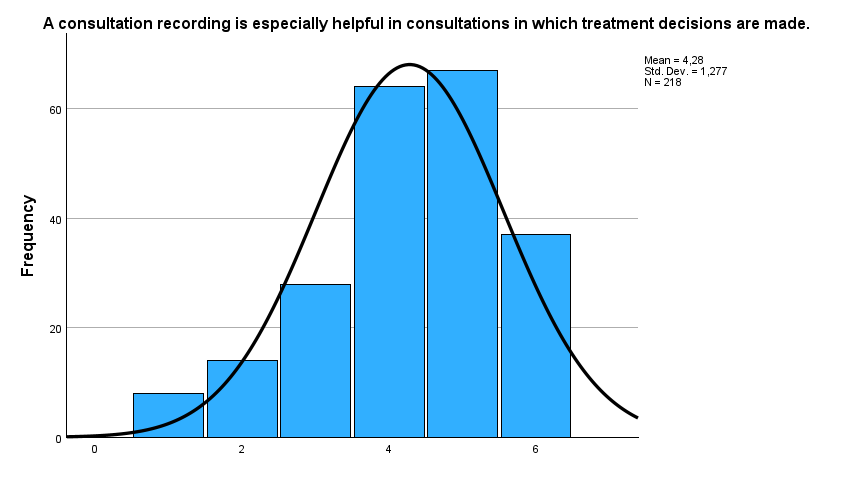

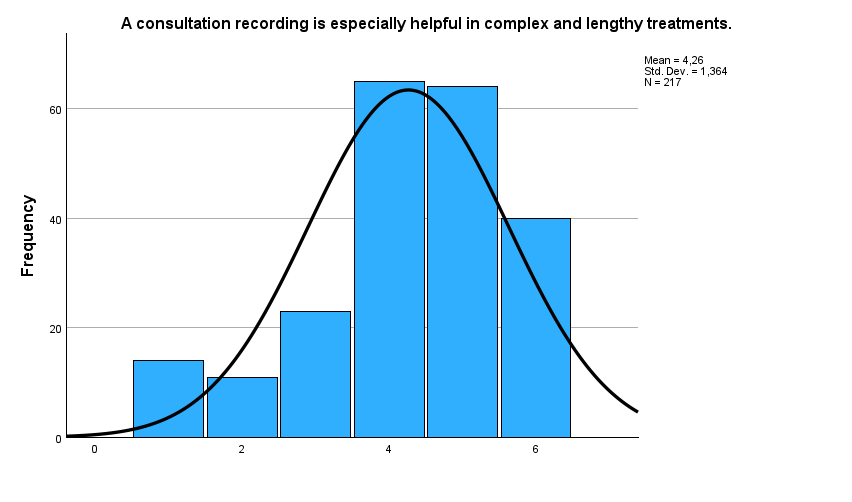

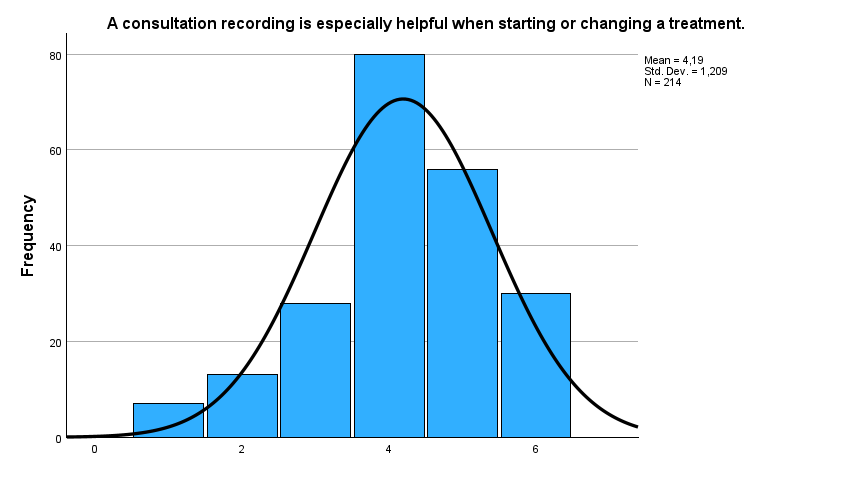

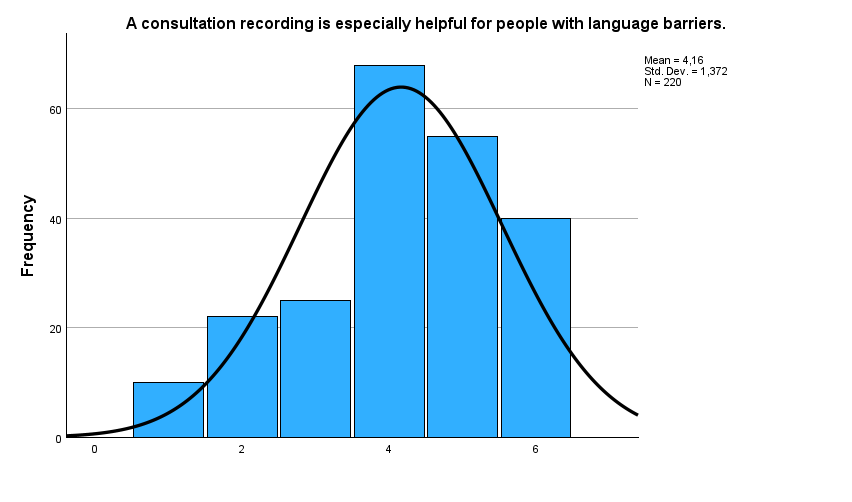

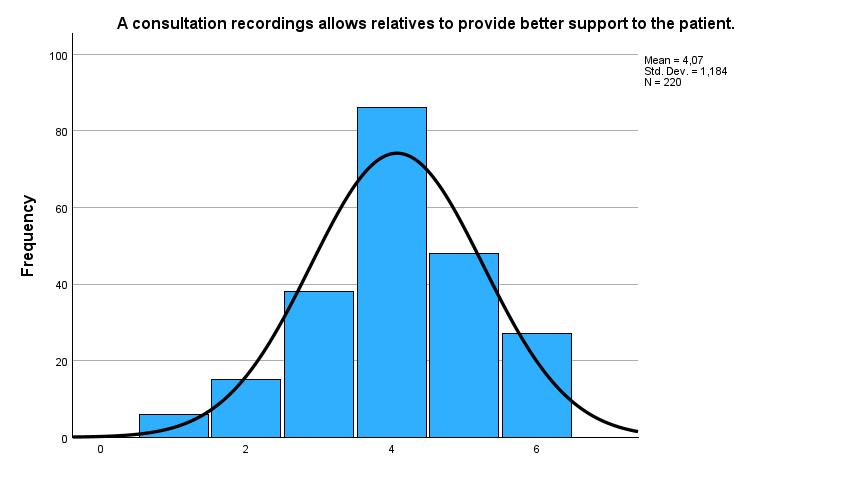

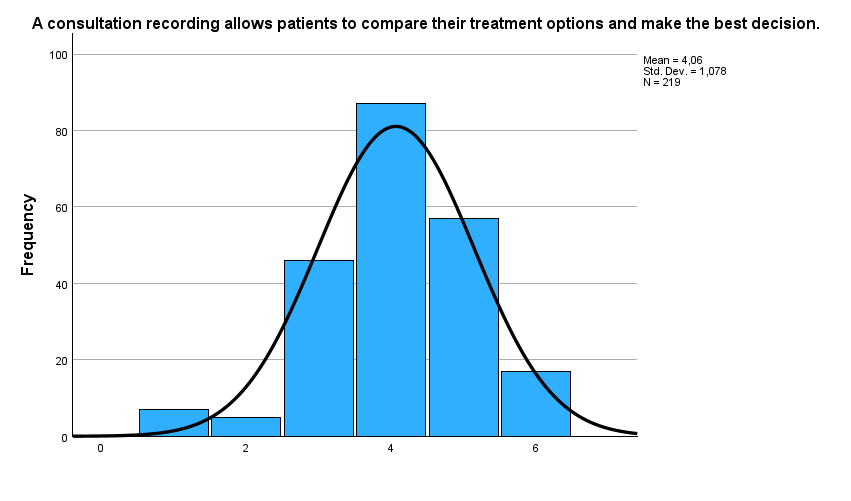

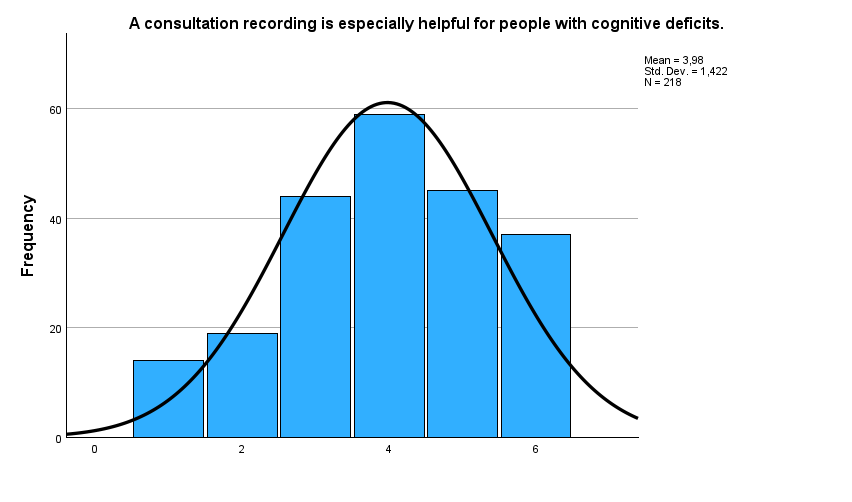

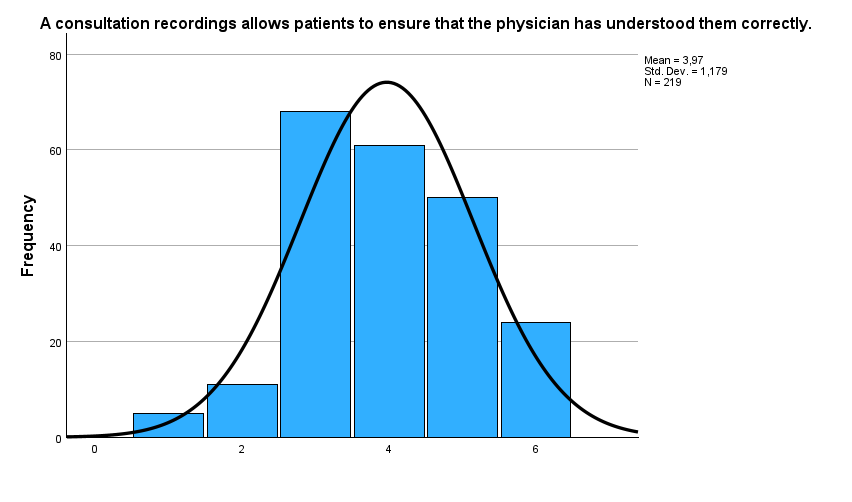

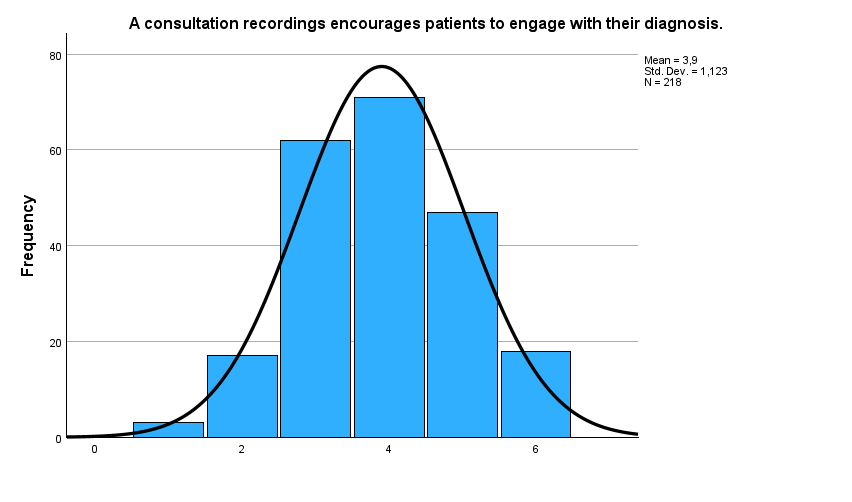

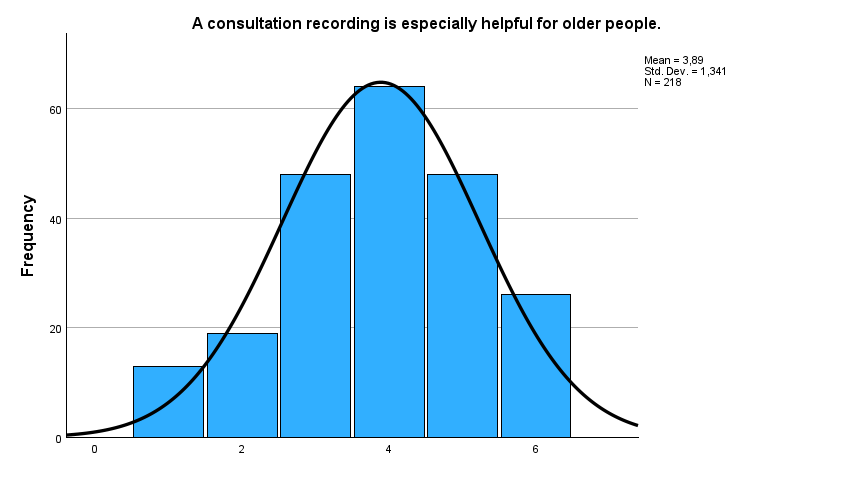

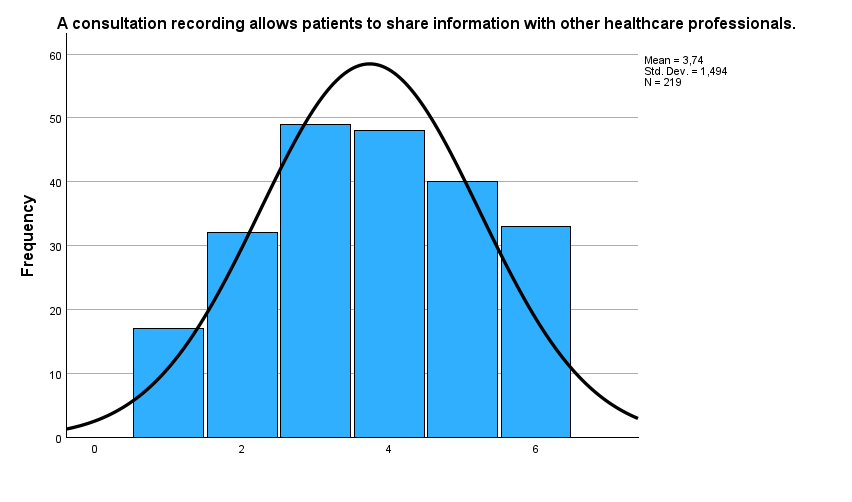

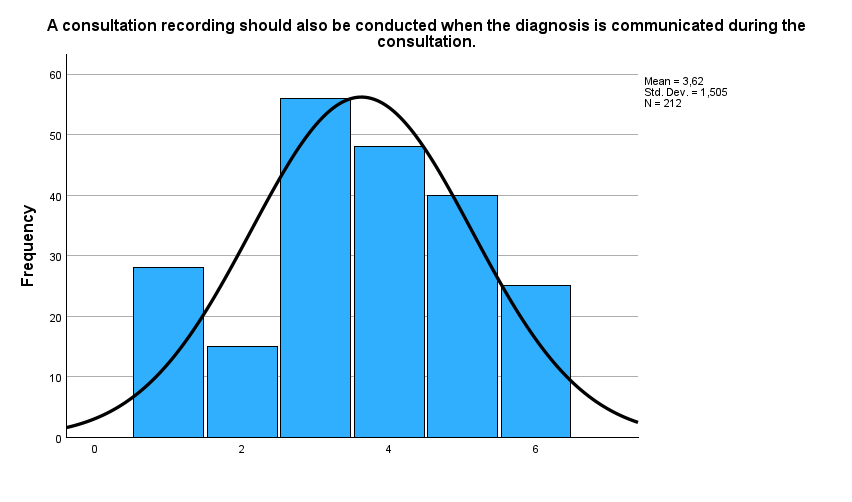

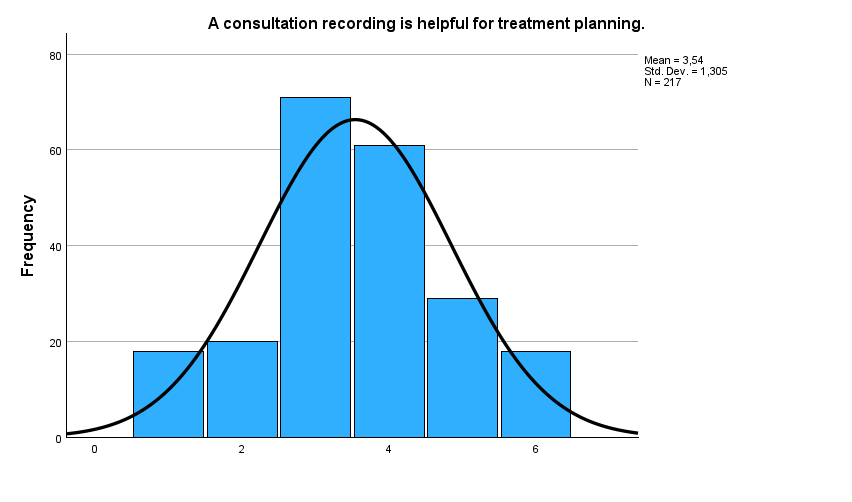

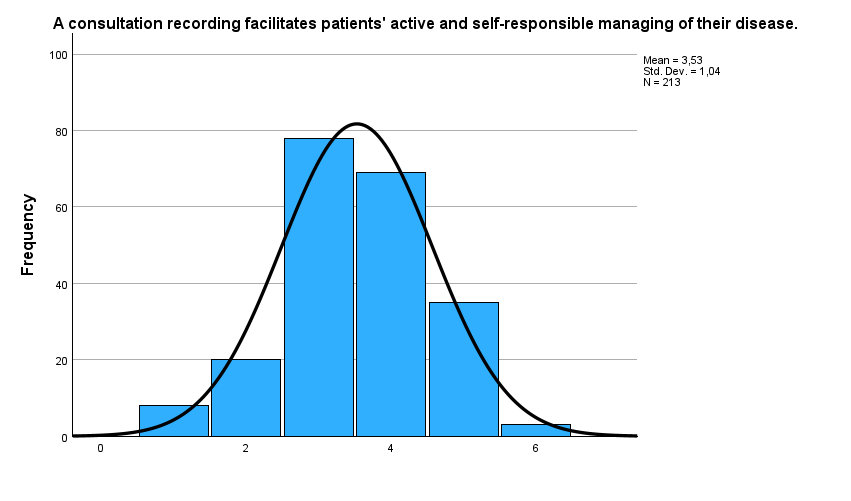

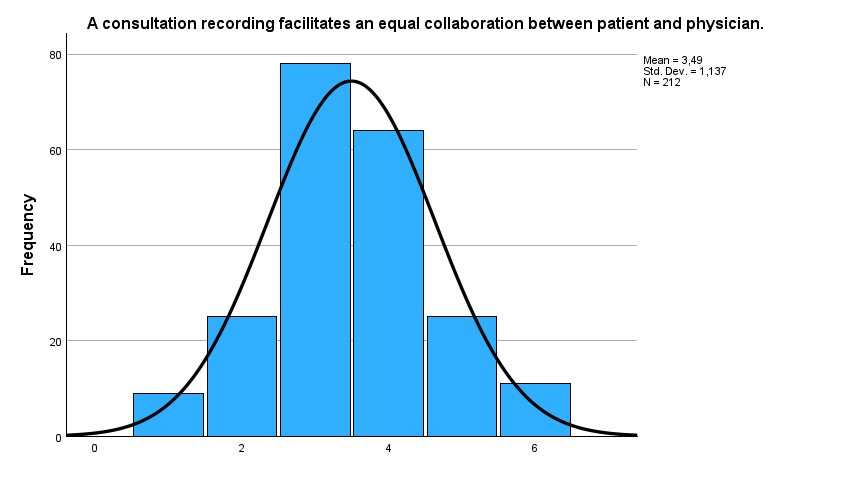

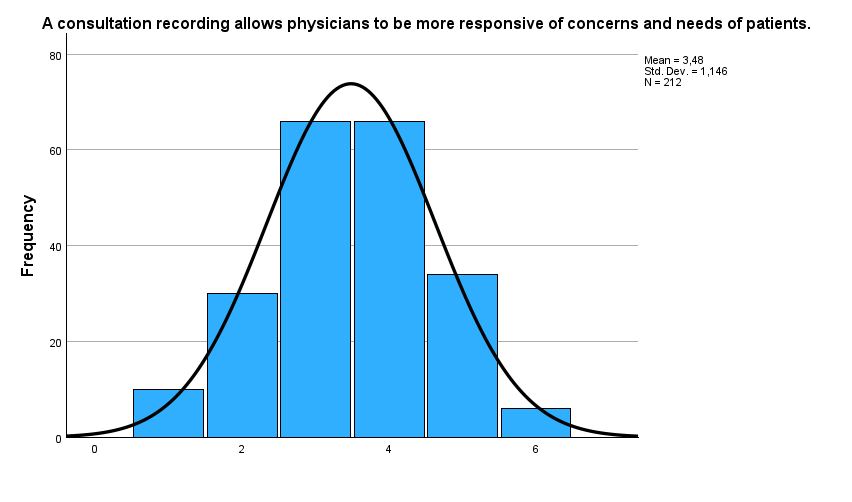

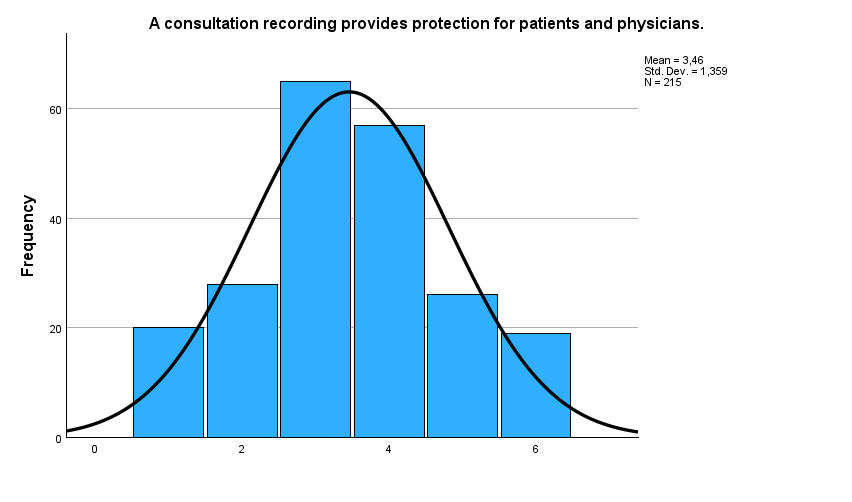

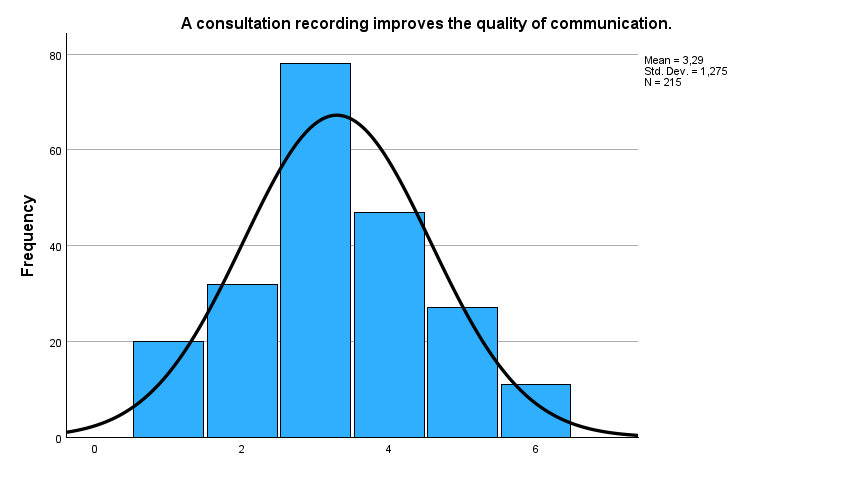

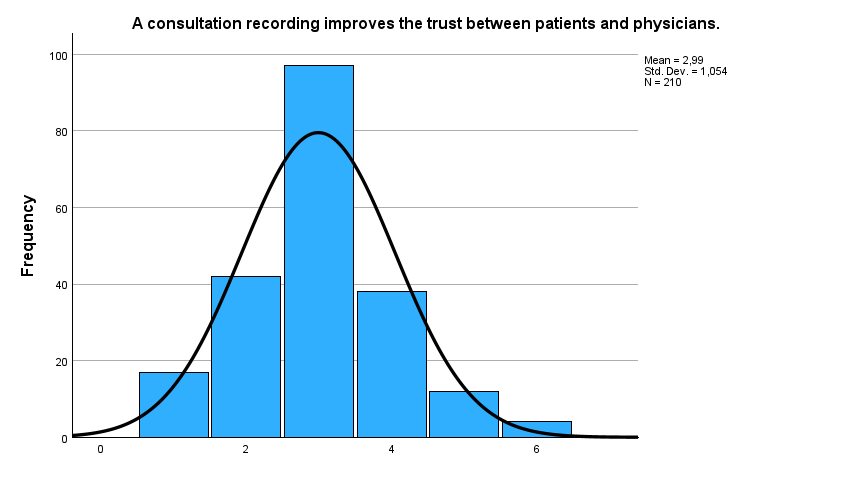

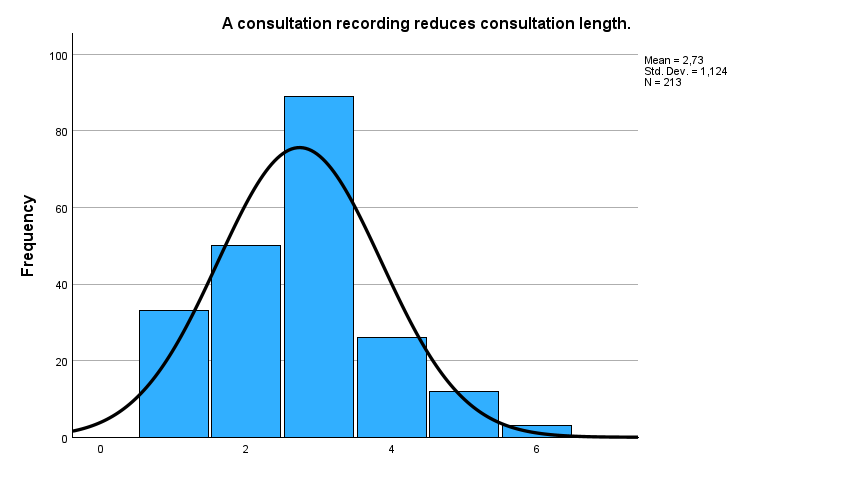

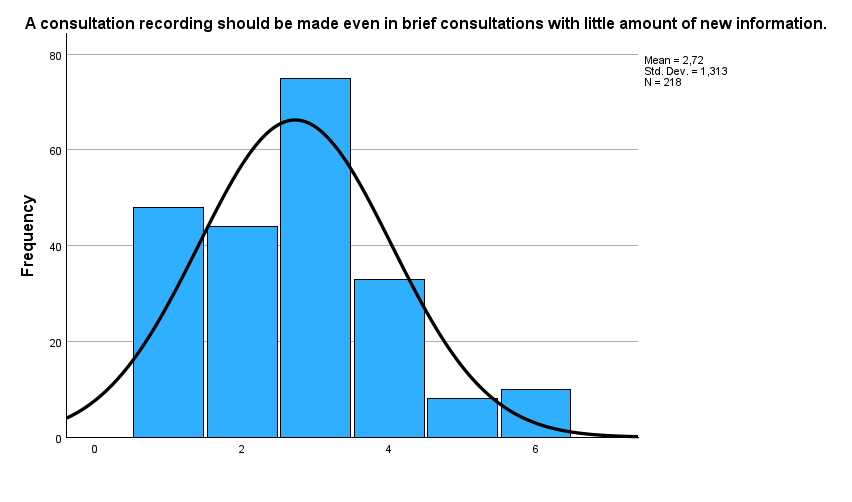
*
